# Putative Role of Neutrophil Extracellular Trap Formation in Chronic Myeloproliferative Neoplasms

**DOI:** 10.3390/ijms24054497

**Published:** 2023-02-24

**Authors:** Dragana C. Marković, Irina S. Maslovarić, Marijana Kovačić, Sanja Vignjević Petrinović, Vesna Lj. Ilić

**Affiliations:** 1 Group for Immunology, Institute for Medical Research, National Institute of Republic of Serbia, University of Belgrade, Dr Subotića 4, 11129 Belgrade, Serbia; 2 Group for Neuroendocrinology, Institute for Medical Research, National Institute of Republic of Serbia, University of Belgrade, Dr Subotića 4, 11129 Belgrade, Serbia

**Keywords:** neutrophil extracellular traps, neutrophils, myeloproliferative neoplasms, inflammation, cell death, apoptosis

## Abstract

Myeloproliferative neoplasms (MPNs) are hematologic malignancies characterized by gene mutations that promote myeloproliferation and resistance to apoptosis via constitutively active signaling pathways, with Janus kinase 2-signal transducers and the activators of transcription (JAK-STAT) axis as a core part. Chronic inflammation has been described as a pivot for the development and advancement of MPNs from early stage cancer to pronounced bone marrow fibrosis, but there are still unresolved questions regarding this issue. The MPN neutrophils are characterized by upregulation of JAK target genes, they are in a state of activation and with deregulated apoptotic machinery. Deregulated neutrophil apoptotic cell death supports inflammation and steers them towards secondary necrosis or neutrophil extracellular trap (NET) formation, a trigger of inflammation both ways. NETs in proinflammatory bone marrow microenvironment induce hematopoietic precursor proliferation, which has an impact on hematopoietic disorders. In MPNs, neutrophils are primed for NET formation, and even though it seems obvious for NETs to intervene in the disease progression by supporting inflammation, no reliable data are available. We discuss in this review the potential pathophysiological relevance of NET formation in MPNs, with the intention of contributing to a better understanding of how neutrophils and neutrophil clonality can orchestrate the evolution of a pathological microenvironment in MPNs.

## 1. Introduction

Philadelphia chromosome (Ph)-negative myeloproliferative neoplasms (MPNs) are heterogenous hematological malignancies characterized by the disruption of hematopoiesis, which implies the over-proliferation of clonal cells from different myeloid lineages, and results in an increased number of mature erythrocytes, leukocytes, or platelets [1,2]. Ph-negative MPN neoplasms may be clinically presented as one of three classical subtypes, namely essential thrombocythemia (ET), polycythemia vera (PV) and primary myelofibrosis (PMF). PV is marked by absolute erythrocytosis, i.e., an increase in red cell mass, and increased numbers of leukocytes and platelets (“trilineage growth”), whilst ET is characterized by the overproduction of platelets associated with bone marrow megakaryocyte hyperplasia, and PMF with the deregulation of the megakaryocyte and granulocyte lineages [1,3,4].

In most MPN cases, genetic defects mainly concern acquired somatic mutations involving Janus kinase 2 (JAK2) [5,6,7,8,9], thrombopoietin receptor (MPL) [10,11], or calreticulin (CALR) genes [12,13], which affect JAK-STAT (signal transducers and activators of transcription) signaling pathways, with JAK2 and STAT5 being the ultimate players, as well as JAK-related pathways PI3K/AKT and Ras/MAPK [5,12,14,15,16,17,18]. JAK2V617F is the most prevalent form of mutation in MPNs with the incidence of mutation as high as ~95% in patients with PV, ~60% incidence in ET patients, and ~50% in patients with PMF [14,19]. Although the cell and molecular signature is distinct between MPN subtypes, these overlap on the signaling pathways and regulatory proteins level, such as STAT, NFκB and/or HIF-1α [20,21,22]. Signaling pathways affected by constitutive JAK2 activation are linked to cell survival, proliferation, and the excessive production of reactive oxygen species via multiple effectors, promoting myeloproliferation, resistance to cell death, and genetic instability [23,24,25,26,27]. The critical complications of the MPN disease are thrombosis, myelofibrosis, and leukemic transformation [28].

The chronic inflammation in MPNs has been described as a pivot for the development and advancement of MPNs from early stage cancer to pronounced bone marrow fibrosis [29,30,31], which may allow for the neoplastic clone to gain a selective advantage over unmutated wild-type cells [32]. According to Hermouet et al. [21], we have still not resolved the issue of whether inflammation-related biological markers and clinical symptoms observed in MPN patients can complement, or succeed, or even precede the acquisition of key mutations harbored by MPN clones [21]. Consequently, the correlative link between inflammation and fibrosis in MPNs is also still not resolved [31]. Different cellular sources in MPNs, in vivo, such as activated leukocytes, platelets, megakaryocytes, and bone marrow stromal cells, which constitute the bone marrow microenvironment or hematopoietic niche, continually release diverse inflammatory mediators, including inflammatory cytokines, chemokines, reactive oxygen species (ROS) and reactive nitrogen species (RNS) [16,29,33,34]. However, mutated (clonal) hematopoietic progenitors and mature blood elements, such as platelets and neutrophils, have been implicated in MPN development as key players underpinning MPN inflammation [21,22,26,32,35,36].

Neutrophils are an essential component of the innate immune response, but they are also a major contributor to inflammation [37]. They display several antimicrobial mechanisms, including degranulation, the production of reactive oxygen species (ROS), phagocytosis, and the formation of neutrophil extracellular traps (NETs) [38,39,40]. The view of neutrophils as terminally differentiated cells without the ability to synthetize proteins has recently been abandoned because in vitro and in vivo studies have revealed the synthesis of cytokines, chemokines, growth factors, and interferons, which is important to the inflammatory process and regulation of immune reactions [41,42,43,44]. Recent studies have revealed that environment can contribute to gene expression profiles of mature neutrophils. Several investigations have disclosed substantial changes in gene expression following LPS induction [45], migration during wound healing [46], activation by phagocytosis, or in the course of apoptosis [47]. These findings define neutrophils as not only translationally active but also transcriptionally active cells, which are responsive to environmental stimuli and capable of a complex series of both early and late changes in gene expression [48]. Accordingly, neutrophils express a broad repertoire of pattern recognition receptors (PRRs) and respond to their stimulation by invariant structural motifs on invading pathogens (pathogen-associated molecular patterns, PAMPs), and/or stress-associated danger signals released from damaged or dying cells (damage-associated molecular patterns, DAMPs) during infection and inflammation [49,50].

In humans, neutrophils are the most prevalent myeloid population in the bone marrow [51,52], and they outnumber all circulating leukocytes [53,54]. Neutrophils have a high daily turnover, with approximately 5–10 × 10^10^ new neutrophils generated each day in the bone marrow from common myeloid progenitors and granulocyte–macrophage progenitors (GMPs) [53,55], recently shown to produce neutrophil-committed proliferative precursors (NeP and pre-Neu) [56,57], which account for 55–60% of bone marrow production [58]; therefore, the balance between the granulopoiesis, storage and egress of mature neutrophils from the bone marrow, intravascular margination, clearance, constitutive death by apoptosis [59] and elimination through phagocytosis [54] is an imperative to maintain neutrophil homeostasis [60].

Apoptosis represents a conserved mechanism of programmed cell death [61] and is generally regarded as non- or even anti-inflammatory. Apoptotic cells are engulfed by phagocytic cells, a process known as efferocytosis, that occurs before the plasma membrane of apoptotic cells becomes porous. This process is marked by the secretion of anti-inflammatory cytokines, such as TGF-β and VEGF [54,62]. Some studies have shown the inflammatory capacity of apoptotic cells when they are not phagocytosed in a timely manner [63], other studies have found that, during apoptosis, the exposure of calreticulin or the autophagy-dependent release of ATP leads to an immunogenic response to apoptotic cells [64,65]. The anti-apoptotic protein myeloid cell leukemia sequence 1 (MCL-1) plays a key role in the regulation of neutrophil apoptosis [66,67]. Nevertheless, cell cycle regulatory proteins, proliferating cell nuclear antigen (PCNA), myeloid nuclear differentiation antigen (MNDA) and cyclin-dependent kinases (CDKs), which in other cell types serve to control proliferation, regulate apoptosis and survival in neutrophils [61,68].

Neutrophil apoptosis is an event in which dying neutrophils continue to exert an immunomodulatory phenotype associated with the resolution of inflammation [69], and against this background, we can think of several facts to support their role in supervising the intensity of the inflammatory response: (1) neutrophils carry distinct types of cargo in neutrophil granules and secretory vesicles, such as myeloperoxidase (MPO), matrix metalloproteinase 9 (MMP9), proteinase 3 (PR3), cathepsin G, neutrophil gelatinase-associated lipocalin (NGAL) and neutrophil elastase (NE) [70,71,72]; (2) neutrophils are translationally and transcriptionally active cells, capable of expressing inflammatory mediators such as cytokines and chemokines [48,73]; (3) they derive two major precursors of all reactive oxygen and nitrogen species - superoxide and NO [39]; (4) neutrophils are important regulators of the hematopoietic niche physiology [52]; (5) neutrophils express a large number of cell surface receptors, which are important for the recognition of pathogens and communication with the inflammatory environment [74]; (6) human neutrophils express key components of inflammasomes, an intracellular protein complex, which serves as a platform for the activation and secretion of proinflammatory cytokines IL-1β and IL-18 [75], although it has been documented that neutrophil serine proteases play a key role in pro-IL-1β processing [76,77]; and (7) they generate neutrophil extracellular traps (NETs), with components that can act as potent pro-inflammatory stimuli [38,78].

In MPNs, the dysregulation of apoptosis is a general phenomenon, due to the constitutive activation of JAK2/STAT, PI3K/AKT and Ras-MAPK/ERK signaling pathways, that modulates the expression of proteins involved in apoptosis [27,79,80]. A number of cytokines, including G-CSF and GM-CSF, that can signal through JAK/STAT pathways, can prolong neutrophil survival in MPNs [48,81,82]. Dysregulated MPN neutrophil apoptosis and their prolonged survival support chronic inflammation, which impacts the development and advancement of MPN diseases from early stage cancer to distinct bone marrow fibrosis [24,30,36,83,84,85].

**Figure 1:**
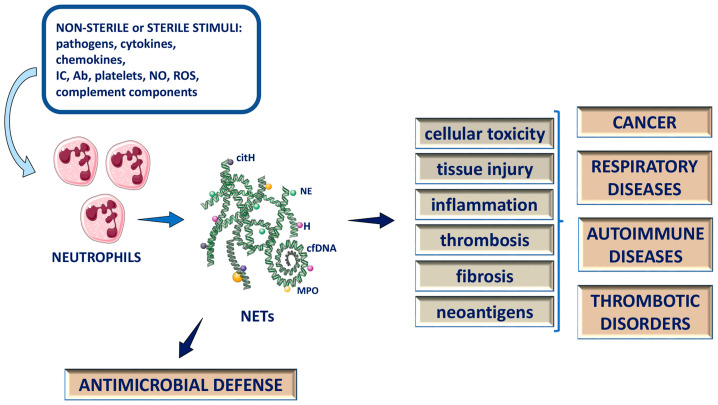
Neutrophil extracellular traps (NETs) in physiology and pathology: antimicrobial defense and deleterious (in a broad spectrum of pathologies) role of NETs. IC: immune complexes; Ab: antibodies; NO: nitric oxide; ROS: reactive oxygen species; cfDNA: cell free DNA; NE: neutrophil elastase; MPO: myeloperoxidase; H: histone; citH: citrullinated histones.

The delay in neutrophil apoptotic cell death and clearance steers them towards secondary necrosis [54,82,86] or NET formation [87], a trigger of inflammation both ways [86,88]. At first, NETs have been approached as a distinct form of programmed cell death and a beneficial innate immune mechanism of host defense against invading microbes (Figure 1), ready to take on responsibility for this vital part of immune defense [38]. Nearly two decades later, neutrophils and NETs, apart from professional inflammatory cells such as myelomonocytic cells [89], were also recognized as crucial contributors to sterile forms of inflammation in several chronic inflammatory conditions [90,91]. The pathological implication of neutrophils and NETs in a diverse pathological milieu emerged in diseases such as autoimmune diseases [92] and cancer [93,94,95], cerebrovascular disease [96], cardiovascular disease, and other acute and chronic diseases [97] (Figure 1). Tripodo et al. [98] demonstrated in animal models that experimental systemic immune activation alters the bone marrow stromal microenvironment, which is linked to NET formation. They used transgenic mice harboring the mutant human nucleophosmin (NPMc) gene driven by a myeloid-specific promoter. The induction of systemic autoimmunity in NPMc transgenic mice elicited modifications in the bone marrow microenvironment and the generation of NETs. NET formation in a proinflammatory bone marrow microenvironment sustained the proliferation of hematopoietic precursors via IL6, SCF and NF-kB activation, and stimulated the progression of dormant NPM1-steered myeloproliferation towards an exacerbated and proliferative dysplastic phenotype [98]. These structural changes and enhanced level of NET structures in the bone marrow were also found in patients with autoimmune disorders and in acute myelogenous leukemia-bearing NPM1 mutation [98]. Overall, their results indicated that bone marrow stromal restructuring, which triggers the formation of NETs in the bone marrow by systemic inflammatory conditions, can complement specific genetic and epigenetic alterations, fostering myeloid malignancy development and progression [98].

In MPNs, even though it seems obvious for secondary necrosis or other forms of lytic neutrophil death to intervene in disease progression by supporting inflammation, no reliable data are available, and when relying on NET studies, one encounters NET data that are predominantly presented in the context of thrombosis as a MPN-associated complication, rather than in the context of lytic NETotic death or vital NET formation and their impact on inflammation and fibrosis in MPN pathology [99,100,101,102,103]. In our recently published paper, we argued that neutrophil death in MPNs is mirrored by defects in apoptosis and listed a set of postulates that could act in support of the statement that the apoptosis of neutrophils supervises the duration and intensity of an inflammatory response, as well as the extent of neutrophil-mediated tissue damage [36]. The findings that MPN neutrophils are primed for NET formation [100,101] and that the signaling pathways and regulatory molecules such as the JAK, STAT, AKT and Raf-MEK-ERK signal transduction pathway—which may regulate both apoptosis and NETs in normal neutrophils or neutrophils under pathological conditions [100,103,104,105,106]—are upregulated in MPNs [5,12,14,15,16,17,18,34] encouraged us to discuss the potential pathophysiological relevance of NET formation in MPNs in this review.

## 2. Neutrophil Extracellular Traps (NETs)

The process of neutrophil extracellular trap (NET) formation is a form of innate immune response which serves to entrap and neutralize pathogens as well as drives the pathophysiological conditions associated with sterile inflammation and autoimmunity [38,90,91,93]. NETs represent the web-like extracellular traps of decondensed DNA strands, decorated with histones and cytotoxic proteins, which are released by activated neutrophils [38].

Two different forms of NET formation have been described: one of them leads to cell death, and the other one is about the cells that retain viability and its effector functions, such as chemotaxis and phagocytosis [107,108,109]. This process is commonly referred to as “NETosis”, since it was initially shown that NET formation unconditionally leads to cell death [38,110]. A more precise conception is that cell lytic NET formation is a special form of programmed cell death (PCD), with DNA strands originating from the cell nucleus [111]. Thus, the Nomenclature Committee on Cell Death (NCCD) 2018 recommended the use of the term NETotic cell death (instead of NETosis) for the process of ROS-dependent regulated cell death associated with NET extrusion [111].

Conversely, neutrophils may expel DNA by the vital formation of NETs [108,112,113]. Vital NET formation, which does not involve plasma membrane damage or cellular lysis, occurs through the nuclear budding and release of vesicles filled with DNA [107,112]. Besides nuclear DNA-containing NETs, vital NETs could be formed through mitochondrial (mt) DNA release [108,113]. Additionally, some studies have shown that both types of DNA, nuclear and mitochondrial, can be concomitantly found in NETs [114]. However, the breakdown of the nuclear membrane is still a hallmark of NET formation for the majority of the researchers in this field [115]. According to the molecular basis of NET formation, two major types of NETs have been reported: NADPH-oxidase (NOX)2-dependent and NOX2-independent NETs [116]. During the assessment of the metabolic requirements for NET formation, Awasthi et al. [117] revealed in their study the importance of glycolysis and the role of lactate accumulation and LDH activity in NET formation by both NOX2-dependent and -independent pathways.

Various stimuli considered to be physiologically relevant can elicit NETs, and include pathogens (bacteria, fungi, protozoa, viruses) [38,118,119,120,121] as well as sterile stimuli, such as antibodies [122], immune complexes [123,124], activated platelets [125,126,127,128], cytokines and chemokines (IL-8, TNF, GM-CSF) [38,108,129,130,131] complement components [132], ROS [104,133] and NO [114,134]. NET induction can be achieved upon the stimulation of specific receptors, such as Toll-like receptors (TLRs), Fc receptors, complement receptors, or cytokine receptors expressed on neutrophils [135,136]. The most commonly used stimuli for NET induction under in vitro conditions are phorbol 12-myristate 13-acetate (PMA) [38,104,119,137] and calcium ionophores, such as calcimycin (A23187) and ionomycin [100,102] or potassium ionophore nigericin [119].

The production of reactive oxygen species (ROS) by NOX2 is considered the biochemical hallmark in the process of NOX2-dependent NET formation (Table 1) and the subsequent neutrophil cell death (NETotic death) [112]. This process requires 2–4 h following the application of an appropriate stimulus [133]. NET formation induced by the PMA, a structural analogue of diacylglycerol, is a protein kinase C (PKC) and NOX2-dependent process [104,137]. PMA directly binds to protein kinase C, which in turn leads to influx of extracellular calcium into the cell and the activation of the NADPH oxidase signaling cascade, resulting in the production of reactive oxygen species (ROS) [104,135,138,139]. Gray et al. [137] demonstrated that conventional PKC, specifically PKCβ, is the predominant isoform responsible for NET formation under these conditions. Several kinases have been implicated downstream of PKC, including c-Raf, MEK (mitogen-activated protein kinase kinases, MAP2Ks), ERK, AKT and phosphoinositide-3-kinase (PI3K) [104,105,111,116,140]. Intracellular ROS trigger the activation and release of myeloperoxidase (MPO), neutrophilic elastase (NE), matrix metalloproteinase 9 (MMP9) and other proteolytic enzymes, from neutrophil granules to the cytosol. In the cytosol, NE, alongside the breakdown of cytoskeletal elements, migrates to the nucleus where it promotes the destruction of a nuclear envelope and, in conjunction with MPO, the decondensation of chromatin [141] (Table 1). The final result is the extrusion of chromatin fibers decorated with histones and cytotoxic granule proteins, plasma membrane rupture and cell death [111,129]. Citrullination is a post-translational protein modification resulting in the conversion of arginine to citrulline and is catalyzed by a calcium-dependent PAD family of enzymes [142]. Peptydilarginine deiminase 4 (PAD4), with cytosolic Ca^2+^ acting as a cofactor, catalyzes the citrullination of histones and participates in chromatin decondensation during NET formation [143,144] (Table 1). However, its involvement remains a matter of debate and appears to depend on the initiating stimulus [111]. Neeli and Radic [145] demonstrated that PMA induces a PKC isoform that suppresses histone citrulination by PAD4. Furthermore, Kenny et al. [119] and Holmes et al. [146] showed that PMA induces primarily uncitrullinated NETs in humans. The complexity of this subject, despite still being arguable, is outlined in the study by Tatsiy and McDonald [131], which shows that the selective inhibition of PAD4 potently prevents PMA-elicited NET formation in human neutrophils.

The NOX2-dependent inducers of NET formation, that act similarly to PMA, are, for instance, *Candida albicans* [118,119], LPS from Gram-negative bacteria *Escherichia coli* [147], Gram-positive bacteria Group B *Streptococcus* (*Streptococcus agalactiae*) [119], pro-inflammatory cytokines [130], nitric oxide [134], oxLDL [148] and immobilized immune complexes [124].

**Table 1:** Regulatory molecules which may drive neutrophil extracellular trap (NET) formation.

Molecules	Roles in NET Formation	References
PAD4	Catalyzes citrullination of histones and participates in chromatin decondensation during NET formation.	[131,143,144]
NADPH-oxidase (NOX)	Production of ROS.	[104,116,133,135]
NOX2-derived ROS	Triggers the activation and release of NE and MPO from neutrophil granules to the cytosol.	[111,141]
Mitochondria-derived ROS	Involved in NET formation. Necessary for spontaneous NET formation of LDG from individuals with SLE. Have a role in the activation of NOX2.	[149,150]
Extracellular ROS (H_2_O_2_, singlet oxygen, HOCl)	NET stimuli.	[131,133,140]
NO	NET stimulus. Activates NOX2, enhances the ROS formation and activates MPO.	[114,134]
NE	Breaks down cytoskeletal elements, promotes the destruction of nuclear envelope and decondensation of chromatin.	[111,141]
MPO	Promotes decondensation of chromatin. MPO, bound to NETs, remains active and generates oxidant species via MPO/H_2_O_2_/Cl^−^ system.	[111,130,141,151]
PKC, c-Raf, MEK, ERK, AKT, PI3K	Involved in NOX2-dependent NET formation.	[104,105,111,116,140]
AKT, p38 ERK1/2, JNK, FAK-2, IKK kinase	Involved in NOX2-independent NET formation.	[106,116,147]
JAK	Potential impact of JAK-STAT signaling on NET formation in MPN patients.	[100,103]
STAT1, STAT3, STAT5 FOXO3, HIF1α, NF-κB, p53, AP-1	“Transcriptional firing” model: transcription in multiple loci and DNA decondensation helps drive NET formation.	[106,152]
Inflammasomes	NLRP3 inflammasome supports NET formation.	[153,154]
CDK	CDK4/6 participates in nuclear envelope disassembling during NET formation.	[87,155]
S1P/S1PR2	Participates in NET formation, relevant for the initiation of chronic liver inflammation and fibrosis.	[156]

The calcium ionophore-induced NOX2-independent NET formation is distinct from PMA-induced NOX2-dependent NETs. This process is fast and mediated by a calcium-activated small conductance potassium (SK) channel member SK3, mitochondrial ROS [116] and PAD4 [119,143,146]. NOX2-independent NET formation is accompanied by ERK1/2, JNK, FAK-2 and IKK kinase activation [106,116]. Unlike NOX2-dependent NETs, NOX2-independent pathway is characterized by a lower level of activation of ERK kinase, while both NOX2-dependent and -independent NET formation require AKT and p38 activity [116] (Table 1).

The NOX2-independent inducers of NET formation, similar to calcimycin (A23187) and ionomycin, are monosodium urate (MSU) crystals (induce NADPH oxidase-independent cell death) [157,158], activated platelets or soluble P-selectin and platelet-derived microparticles [125,126,127], diverse microorganisms [151,159], complement proteins [132], GM-CSF, TNF-α [131] and soluble immune complexes [123].

Even though the NOX2-dependent and NOX2-independent mechanisms of NET formation still stand out as the most common, from 2004, when Brinkmann et al. [38] described NET formation for the first time, numerous up-to-date studies have shown that NET release can occur via distinct pathways and molecules depending on the type and concentration of stimulus that triggers the process, as well as on the duration of stimulation required for NET release, as shown in in vitro studies [115,119,146,160]. In relation to generalized schemes, the differences in the presence/absence and the significance of molecules such as MPO [119,124,151], specific kinases [113,131,147], citrullinated histones [111,119,146], PAD4 [119,131] or the relevance of processes such as the citrullination of proteins in general [146], mobilization of intracellular and extracellular calcium pools [139] and the requirement of transcription for NET formation [106,161] are reported.

As opposed to the necessity of NET formation in innate immune response against bacterial, viral and other pathogens, NETs have recently been acknowledged to express a deleterious effect on various sterile inflammatory conditions such as cancer [93,94], autoimmune diseases [149,162], diabetes [163] and thrombotic disorders [164]. However, even when the disease is caused by a pathogen, the formation of NETs can be harmful, such as in the case of COVID-19 pathology, wherein excessive NETs are associated with increased neutrophil activation, the development of high levels of circulating cell-free DNA (cfDNA), “cytokine storm” and coagulopathies [121,165,166]. Although sporadic, there is a report that, under some conditions, NETs may have a beneficial role. Schauer et al. [167] reported, in chronic gout, the formation of aggregated NETs, which promote the resolution of neutrophilic inflammation by degrading cytokines and chemokines and disrupting neutrophil activation.

However, most of the data indicate that NET components, such as circulating cfDNA, extracellular histones and granule proteins, are cytotoxic, pro-inflammatory and promote tissue injury, fibrosis and coagulation [87,97,168,169,170,171] (Figure 1). Several chronic inflammatory diseases, regardless of the cause (sterile or non-sterile) of inflammation, are marked by a continued influx of neutrophils and sustained NETs release. In respiratory chronic diseases such as cystic fibrosis (CF) and chronic obstructive pulmonary disease (COPD), persistent NET formation, which is associated with inflammation and disease severity, was found [87,172,173]. Neutrophils and NETs may play a critical role in autoimmune diseases such as systemic lupus erythematosus (SLE) and rheumatoid arthritis (RA) [92,174,175]. Villanueva et al. [92] reported that a distinct low-density granulocyte (LDG) population found in SLE patients demonstrated their tendency for spontaneous NET formation and the upregulation of proteins and enzymes expression implicated in NET formation and autoimmunity. There is growing evidence that these NETs also expose double-stranded DNA, an autoantigen implicated in lupus pathogenesis, through the induction of IFN-α synthesis by plasmacytoid dendritic cells [92,176]. It is shown that RA neutrophils have activated NF-κB signaling, leading to delayed apoptosis [177]. The authors further showed that RA synovial fluid (SF) neutrophils have an altered phenotype, increased expression of chemokines, FcγR1 and MHC II, decreased apoptosis and increased ROS and NET production [175]. In cancer development, when the body’s inflammatory response to tissue damage caused by either physical or ischemic injury, or infection, becomes chronic, cell mutation and proliferation can result, creating an environment that favors cancer evolvement and progression [178]. Demers et al. [95] showed, in murine models of chronic myelogenous leukemia (CML), breast, and lung cancer, that a systemic environment is created in cancers, which can induce an increase in peripheral blood neutrophils, predisposed to NET formation. It has been reported that NETs induced by a pro-inflammatory trigger can mediate the proteolytic remodeling of the laminin, matrix component, to divulge a novel epitope that triggers the proliferation of quiescent cancer cells via integrin activation [94,179]. Additionally, it is shown that NET components promote cancer-associated thrombosis and cancer cell proliferation, migration and metastasis [93,94,95].

### NET Formation in MPNs

**Figure 2:**
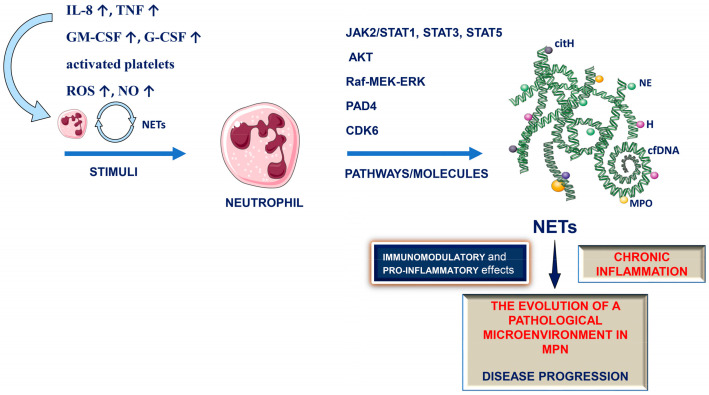
Stimuli, signaling pathways and regulatory molecules which may drive neutrophil extracellular trap (NET) formation in myeloproliferative neoplasms (MPNs), and the potential pathophysiological relevance of NET formation in MPNs. IL-8: interleukin-8; TNF: tumor necrosis factor; GM-CSF: granulocyte macrophage-colony stimulating factor; G-CSF: granulocyte-colony stimulating factor; ROS: reactive oxygen species; NO: nitric oxide; JAK2: Janus kinase 2; STAT: signal transducers and activators of transcription; AKT: protein kinase B; Raf: rapidly accelerated fibrosarcoma kinase; MEK: mitogen-activated protein kinase kinases, MAP2Ks; ERK: extracellular signal-regulated kinase; PAD4: peptidylarginine deiminase 4; CDK6: cyclin-dependent kinase 6; cfDNA: cell-free DNA; NE: neutrophil elastase; MPO: myeloperoxidase; H: histone; citH: citrullinated histones. ↑ denotes increased expression.

Various stimuli that could activate NET formation are present in MPNs: the elevation of cytokines and chemokines, such as IL-8, TNF, G-CSF, GM-CSF [33,180,181], activated platelets [35], elevated ROS and NO [34] (Figure 2). Most importantly, members of the STAT family of transcription factors (STAT1, STAT3 and STAT5) were recently reported as important for both PMA- and A23187-induced NET formation [106]. In addition, it is shown that PAD4 protein expression was increased in neutrophils from PV patients with JAK2V617F mutation, compared to healthy controls [100]. 

Signaling pathways and regulatory molecules, such as JAK, STAT, AKT and Raf-MEK-ERK, which may regulate NET formation in normal neutrophils or neutrophils under pathological conditions [100,103,104,105], are upregulated in MPNs, as components of JAK2-STAT signaling pathways [5,12,14,15,16,17,18,34]. Furthermore, MPN neutrophils are characterized by the upregulation of JAK target genes, regardless of JAK2 mutational status [17]. Therefore, it is reasonable to speculate that clonal neutrophils are an important source of NET generation in MPNs (Figure 2).

The information on NET formation in MPNs almost exclusively comes from the studies on isolated neutrophils, either stimulated or unstimulated [99,100,101,102,103]. The only in situ up-to-date study regarding NETs in MPNs was observed in a mouse model, where an increase in citrullinated histone H3 (H3cit) expression was demonstrated in the lung tissue sections of mice expressing the JAK2V617F mutation [100]. Only five studies to date [99,100,101,102,103] have investigated NETs in MPNs, all of which were in the context of NETs being involved in the pathogenesis of thrombosis, which is determined as a main cause of morbidity and mortality in MPN disease [182,183]. In addition, one review article [183] summarized the literature linking NETs and thrombosis in MPN settings. In the study by Guy et al. [101], the ex vivo analysis of NET formation showed that unstimulated neutrophils from patients with MPNs are more prone to form NETs than controls. On the contrary to these findings, the study by Marin Oyarzún et al. [99] did not find enhanced NET formation by unstimulated JAK2V617F neutrophils. Under stimulation, a study by Marin Oyarzún et al. [99] revealed that neutrophils from patients with MPNs do not show enhanced NET formation in vitro when stimulated with proinflammatory cytokines compared to controls, while with PMA as a stimulant, MPN neutrophils showed impaired NET formation, particularly in patients with myelofibrosis. Conversely, Wolach et al. [100] demonstrated that the JAK2V617F expression is associated with increased NET formation in response to neutrophil stimulation with PMA or ionomycin in both neutrophils from MPN patients and from JAK2V617F mice. They demonstrated that neutrophils from patients with MPNs are primed for NET formation, an effect blunted by ruxolitinib, a JAK inhibitor, that selectively inhibits the JAK1 and JAK2 protein kinases. Furthermore, they reported that the inhibition of JAK2 signaling may reduce thrombosis in MPNs through the inhibition of NET formation. Namely, the treatment with ruxolitinib abrogated NET formation in vitro and decreased thrombosis in JAK2V617F mice in vivo. Craver et al. [102], in contrast with the study by Marin Oyarzún et al. [99], showed that PMA rapidly induces NET formation in neutrophils from MPN patients, but without differences compared to controls. Additionally, they demonstrated that N-acetylcysteine (NAC), a free radical scavenger, reduced PMA-induced NET formation in both normal and MPN neutrophils, and inhibited thrombosis in a murine model of MPNs. Concerning the role of platelets in NET formation, the results found by Craver et al. [102] suggested that the interaction between platelets and neutrophils in MPNs may play a crucial role in promoting NET formation. In another Philadelphia chromosome-negative MPN cohort study, Schmidt et al. [103], by quantifying in vitro NETs induced by the treatment of neutrophils with ionomycin, showed: (1) a clearly higher level of NET formation in MPN patients than in healthy donors; (2) positive correlation between somatic mutations in the JAK2 V617F and CALR genes and increased NET formation; and (3) that NET formation is not linked to either thrombosis or to classical risk factors for thromboembolic complications. However, the study showed that the JAK2 allelic burden was not associated with the rate of NET formation, and ultimately it did not seem likely that the genetically driven NET formation would translate in the clinical endpoint, such as thrombosis in MPNs, which put their results in contrast to the effects seen by Wolach et al. [100]. Since the experimental setup for assessing the potential impact of JAK-STAT signaling on NET formation was not ideal according to the authors [103], the function of JAK-STAT signaling on NET formation remains elusive and needs to be considered in detail. However, these results do not come as a surprise since NETs themselves induce more NETs in neutrophils [184], which can be both malignant and normal cells in MPNs, in vivo [21]. Thus, the JAK2 allelic burden does not necessarily have to be correlated with the rate of NET formation under in vitro conditions. In agreement with this are the findings, in murine models of CML, showing that not only the transformed neutrophils but also the normal neutrophils are primed to generate NETs [95]. Anyhow, the authors themselves [103] further advised caution when interpreting the currently available data on NETs and its potential clinical impact on MPNs since many studies, including their own, have used the model of in vitro triggered NET formation where the endogenous trigger of NETs is not known and therefore, may not sufficiently reflect the process of NET formation in vivo. Regarding the plasmatic biomarkers of NETs, the concentration of plasmatic free DNA and MPO-DNA complexes was significantly higher in patients with MPNs than in the controls [101], while Marin Oyarzún et al. [99] and Schmidt et al. [103] demonstrated elevated levels of circulating nucleosomes in patients with MPNs. In favor of the pathogenic role of NET formation in the occurrence of thrombosis, Guy et al. [101] showed that MPO-DNA complexes were higher in patients with a history of thrombosis compared to those without thrombosis.

The overall results on NET formation presented above [99,100,102,103] underline the potential of diverse stimulants (cytokines/PMA/ionomycin) to induce NETs in MPN patients, although the methods of evaluating the NET formation are also of importance [115,146]. We may draw a conclusion that unstimulated neutrophils from patients with MPNs are more prone to form NETs than controls [100,101] and that upon stimulation with PMA as well as with ionomycin, MPN neutrophils exhibited elevated NET formation [100,103], although these results are contradictory according to Marin Oyarzún et al. [99]. In addition, the presented results showed a pathogenic role of NET formation in the occurrence of thrombosis in MPNs [100,102].

## 3. Points to Reconsider in Further Research

Uncertainties about NETs in general, as well as regarding MPNs, still require our undivided attention, concerning different types of NETs, pathways and mechanisms of formation, methods for the assessment of NET formation and the role of NETs in health and diseases. Here, we will present some of the aspects which we consider to be of importance in this context.

### 3.1. NETs in MPNs in the Context of Inflammation, Fibrosis and Thrombosis

The main components of NETs are cell-free nuclear and/or mitochondrial DNA, namely cfDNA. NET-derived cfDNA can be highly oxidized pro-inflammatory molecules [149,179,185] that can act as damage-associated molecular patterns (DAMPs) [186,187]. The main cfDNA recognition pathways are leading from Toll-like receptors (TLR) 3-7-8-9 [188]. Among these receptors, TLR9, present in intracellular endosomal vesicles, but equally at the extracellular surface of neutrophils, is implicated in the recognition of small-sized oligonucleotides containing demethylated CpG motifs [188]. The binding of cfDNA to TLR9 is accompanied by cytokine secretion via the action of NF-kB [188,189]. Mitochondrial DNA is packaged into mtDNA–protein complexes called nucleoids. The protein commonly recognized as a core component of the mammalian nucleoid is the mitochondrial transcription factor A (TFAM), which is abundantly expressed [190,191]. Mitochondrial DNA is more susceptible to oxidative damage than nuclear DNA, as it lies close to ROS production sites [149,162]. Furthermore, mtDNA is a more potent pro-inflammatory stimulus than nuclear DNA [169,192], given that it is enriched in unmethylated cytosine-phosphate-guanine (CpG) dinucleotide motifs, which are highly potent TLR-9 ligands [192,193]. TLR9 response to CpG DNA initiates the production of proinflammatory cytokines and type I IFN [194]. CpG dinucleotide repeats also trigger IL-10 secretion and transforming growth factor beta (TGF-β) release [171]. Although protective anti-inflammatory signaling is important for the resolution of inflammation, on the other hand, TGF-β could drive unrestrained fibrotic response [54]. Besides its pro-inflammatory effect, NET-derived cfDNA is a strong activator of thrombotic disorders, since both mitochondrial and nuclear cfDNAs carry procoagulant and platelet-stimulating potential [169].

In addition to DNA, NETs contain more than 80 different molecules, such as: histones, serine proteases (NE, AZU1, PR3, CatG), cytoskeletal proteins, antimicrobial peptides and proteins (MPO, S100A8/9, α-defensin, peptide LL-37) and iron-binding proteins (lipocalin 2, lactoferrin), all of which have immunomodulatory and pro-inflammatory properties in the extracellular space [78,195]. Extracellular histones, vital elements of NETs with bactericidal action, are cytotoxic, act as inducers of thrombosis, increase ROS production and recruit neutrophils, creating a loop in favor of NET production [196]. In the extracellular space, histones act as DAMPs by activating Toll-like receptors (TLRs), promoting proinflammatory cytokine pathways and altering phospholipid membrane permeability [197]. The most examined neutrophil granule proteins in the context of NET formation are MPO and NE. In the extracellular environment, MPO, bound to NETs, remains active and can damage the extracellular matrix (ECM) through oxidants [78,198]. NE can also cleave ECM components and has been identified as an active participant in some fibrotic-related diseases [199]. Additionally, NE elicits the release and activation of TGF-β [200]. In the tumor environment, NET-associated granule proteins can cause tissue injury and contribute to tumor metastasis by upregulating protease activity, such as MMPs and NE, thereby promoting tumor progression [170,171]. NETs also contain calprotectin (CLP), a heterodimer formed by S100A8 and S100A9 [201]. One of the most important pro-inflammatory features of S100A8/A9 is its role as DAMP, an agonist for the TLR4/MD2 complex [202].

Human neutrophils express the key components of inflammasomes, an intracellular protein complex, which serves as a platform for the activation and secretion of proinflammatory cytokines IL-1β and IL-18 [75]. Recently, a group of authors evaluated the link between inflammasomes and NET formation in a sterile environment [153,154] (Table 1), since it was observed that NET formation and the inflammasome assembly are associated with similar disorders in humans [153]. Their data point to an important connection between NLR family pyrin domain containing 3 (NLRP3) inflammasome and NETs, where the formation of the NLRP3 inflammasome supports NET formation, while PAD4 regulates NLRP3 inflammasome assembly in neutrophils [153]. An inhibitor of NLRP3 reduces the toxic effects of NETs, and PAD4 inhibitors are proposed to improve inflammasome-driven human disorders and diseases [153]. Furthermore, Wang et al. [154] revealed a new role for NETs released by neutrophils in the activation and regulation of inflammasomes during the progression of non-small cell lung cancer (NSCLC), associated with the activation of NF-κB.

Furthermore, Dömer et al. [184] challenged human primary neutrophils in vitro with NETs as a stimulus and showed that NETs generate more NET formation through the production of ROS by the NADPH oxidase-dependent pathway. As such, their results demonstrated the proinflammatory role of NETs by activating the effector functions of human neutrophils that can lead to detrimental outcomes in chronic inflammation supporting the biological relevance of this process. NETs also influence the inflammatory immune response via modulatory effects on other cells, such as macrophages, dendritic cells and T-lymphocytes [184,203,204].

Investigating pulmonary fibrosis, Chrysanthopoulou et al. [168] pointed out that NETs are not only involved in inflammation but also in fibrosis, and that NET components, in conjunction with a possible defect in NET clearance, may have an impact on fibroblast activation, contributing to disease progression towards fibrosis. They described the critical role of histone citrullination and neutrophil autophagy in the fibrotic process, via the regulation of NET release. Importantly, they showed NET deposition in human lung fibrotic biopsies. Although no intact neutrophils were evident in the biopsies, the NET deposition was detected, as observed by the extracellular localization of MPO and neutrophil elastase. These data suggest that the remnants of neutrophil activation such as NETs components may drive the inflammatory and fibrotic processes in the tissues affected by chronic inflammation [168]. In cystic fibrosis (CF), neutrophils are essential inflammatory cells in the lungs of patients. Decreased spontaneous neutrophil apoptosis and increased NET formation that can promote inflammation have been described in CF disease [87]. The authors suggested the inhibition of NET formation as a driving force towards neutrophil apoptosis, neutrophil disposal, and the subsidence of inflammation.

The NET-dependent regulation of inflammation has been documented predominantly under sterile conditions, such as cancer [93,94], autoimmune diseases [149,162], diabetes [163], thrombotic disorders [164], atherosclerosis [203] and pulmonary fibrosis [168]. However, in MPNs, when relying on the NET studies, one can come across the NET data predominantly presented in the context of thrombosis [99,100,101], as an MPN-associated complication, and not in the context of NET formation with an impact on chronic inflammation and fibrosis in MPN pathology, which are also among the central features of MPNs [31,205]. Nevertheless, the correlative link between inflammation and fibrosis in MPNs is still a lingering issue, recently defined as “Still a burning question” [31]. Furthermore, Tripodo et al. [98] showed, on transgenic mice harboring mutant human NPMc gene driven by a myeloid-specific promoter, that a proinflammatory bone marrow microenvironment supports NET formation and discovered a novel role for NETs in stimulating hematopoietic precursor proliferation. Their results indicated that bone marrow stromal restructuring, which triggers the formation of NETs in the bone marrow by systemic inflammatory conditions, can complement specific genetic and epigenetic alterations, fostering myeloid malignancy development and progression [98]. Regarding inflammasomes, Zhou et al. [206] showed the high expression of inflammasome-related genes (NLRP3, NF-κ β1, CARD8, IL-1β and IL-18) in bone marrow cells from MPN patients and the association between the increased expression and JAK2V617F mutation, white blood cell counts and splenomegaly. Another inflammasome important in the context of MPN disease is the absent in melanoma 2 (AIM2), which recognizes double-stranded DNA (dsDNA) [207,208], and Liew et al. [209] identified AIM2 as a downstream target of JAK2V617F in hematological cell lines expressing JAK2V617F. The induction of JAK2V617F leads to an inflammatory response [209], which is consistent with the studies demonstrating the involvement of IL-1β in the development of myelofibrosis in a JAK2V617F mouse model [210]. However, the link between inflammasomes and NETs in MPNs has not been addressed until today. In this regard, and in line with the findings presented above, it is fruitful to continue systematic research on NET formation in MPNs.

### 3.2. Regulatory Molecule Survivin in the Context of NET Formation in MPNs

By clarifying the inclination of bone marrow neutrophils to NET formation in MPN patients, we could make a difference in dealing with chronic inflammation as a pivot for the development and advancement of MPNs. In this regard, regulatory molecule survivin is of particular importance. Immature neutrophils express survivin, an inhibitor of apoptosis, whereas in mature neutrophils, survivin is absent or only marginally expressed, but can be induced using growth factors such as GM-CSF and G-CSF [211], which achieve their signaling via JAK/STAT pathways, after binding to specific receptors [74]. In addition, high levels of survivin were expressed in mature neutrophils under inflammatory conditions in vivo [211]. Furthermore, survivin overexpression was detected in the bone marrow cells of MPN patients [212,213]. Keerthivasan et al. [214] identified a novel role of survivin during the terminal stage of the differentiation of red blood cells, in erythroblast enucleation. They also demonstrated that survivin overexpression is sufficient to induce the enucleation of murine erythroleukemia (MEL) cells. It is not known whether survivin expression similarly enhances NET formation, specifically that of vital NETs [112]. Another question is whether immature neutrophils are the primary NET-forming cells due to expression of survivin [112]. In this regard, it should be pointed out that almost all major granule proteins involved in NET formation, such as neutrophil elastase, myeloperoxidase and cathepsin G, are located in azurophilic granules whose formation begins at the promyelocyte stage [72,215]. ROS production starts at the myelocyte stage with the formation of specific granules [72]. Therefore, the ability of neutrophils to form NETs is acquired at the mitotic pool stage, in the granulopoietic compartments of neutrophils within the bone marrow. Although the receptors for various inflammatory stimuli are generally expressed at low levels in promyelocytes, myelocytes and metamyelocytes, and become highly expressed only in mature neutrophils [216], some NETs stimulators such as hydrogen peroxide, which is a membrane permeable molecule, can directly induce NETs in neutrophils [131,133]. What this could mean in the context of MPNs, since the MPN bone marrow stem cell niche is a site of chronic inflammation, with commonly increased cells of myeloid lineage, including neutrophils [1,29], remains to be determined.

### 3.3. Pathways and Molecules That Regulate Both Apoptosis and NET Formation

Several independent studies have shown the presence of pathways or molecules that regulate both apoptosis and NET formation, in a sense of suppressing apoptosis and supporting NET formation, under either normal or pathological conditions [87,104,105,156].

In the search for an effective therapeutic strategy for fatty liver disease, a group of authors [156] suggested, in their findings, an important role of neutrophils and NET formation in the initiation of chronic liver inflammation. The need to clarify the underlying molecular mechanisms of regulating neutrophil function prompted these authors to investigate a pleiotropic lipid mediator, sphingosine 1-phosphate (S1P), one of the potent regulators of cellular processes important for inflammation response [217]. A substantial increase in S1P levels and its important role in the exacerbation of liver injury caused by multiple etiologies have been demonstrated by the authors in their earlier studies [218,219]. S1P is a ligand for five specific G protein-coupled S1P receptors (S1PR) 1–5 that activate diverse downstream signaling pathways [156]. They showed that the S1PR2 knockdown ameliorates liver inflammation and fibrosis by inhibiting NET formation in vivo, which is why they speculated that S1P mediates the switch of neutrophil spontaneous apoptosis to NET formation in in vitro settings (Table 1). This process is dependent on S1PRs coupling to different G-α protein subunits, and it regulates NF-κB, PI3K/Akt, Erk, p38 MAPK and ROS signaling pathways [156].

Furthermore, Douda et al. [105] are emphasizing PMA as an activator of AKT with the end result of NET induction. They found that NOX2-mediated NET formation is dependent on AKT activation, and that the suppression of AKT prevents NET formation and allows the induction of apoptosis.

The study by Hakkim et al. [104] showed that the Raf-MEK-ERK pathway, involved in NET formation through the activation of NADPH oxidase, leads to the upregulation of anti-apoptotic protein Mcl-1. This suggests that the Raf-MEK-ERK pathway inhibits apoptosis to allow NET formation [104].

It is reported that some members of the cyclin-dependent kinase (CDK) family regulate both apoptosis and NET formation in neutrophils [87]. CDK4/6 isoforms are enzymes of critical importance for cell-cycle progression. In addition, CDK6 is a direct regulator of transcription in both kinase-dependent and -independent aspects, interacting with a range of transcription factors, including the STAT family [220]. In neutrophils, CDKs act as neutrophil-specific pro-survival regulatory factors [61,68]. These cell-cycle proteins are localized in the nucleus of proliferating cells, while in mature neutrophils, they are located in the cytoplasm [61,68]. Amulic et al. [155] demonstrated that CDK4/6 is required for NET formation (Table 1). Furthermore, they showed that CDK6 transiently enters the nucleus after the stimulation of human neutrophils by PMA. CDK6 inhibition blocks NET formation, and neutrophils from a CDK6^−/−^ mouse strain are impaired in NET production [155]. CDK4/6 cell-cycle proteins most likely participate in nuclear envelope disassembling during NET formation [155]. Delayed neutrophil apoptosis is described in cystic fibrosis (CF) lung disease [87], a non-sterile inflammation, where the authors demonstrated that CF neutrophils form more NETs because of disturbed apoptosis and suggested CDK inhibitors to overturn NET formation in CF and drive cells towards apoptosis and inflammation resolution. In addition to this potential role of CDK6 to regulate both apoptosis and NET formation in neutrophils, it was also observed, in JAK2V617F progenitor cells, that the CDK6 protein acts as a transcriptional regulator of NF-κB signaling, apoptosis and malignant stem cell activation, which mainly illustrates the kinase-independent and non-catalytic functions of CDK6 in MPN pathogenesis [220]. The absence of CDK6 mitigates clinical symptoms and extends the survival of JAK2V617F transgenic mice [220]. The presented data identify the key role of CDK6 inhibition in prevailing pro-inflammatory signaling by targeting the inflammatory resolution switch, driving apoptosis and phagocytic clearance of apoptotic cells and by regulating the malignant stem cell quiescence [220]. Even though the CDK6 activity and NET connection has not yet been demonstrated, directly in MPNs, these data provide the rationale for the therapeutic evaluation of CDK6 inhibition in MPN disease [61,68,155,220].

Based on the studies presented above, although the key molecules connecting neutrophil apoptosis to NET formation, in a sense of suppressing apoptosis and supporting NET formation, were not demonstrated in MPNs, it is reasonable to speculate that clonal neutrophils, with constitutively activated signaling pathways leading to deregulated apoptotic machinery, are an important source of NET generation in MPNs. We hope that future work will clarify this hypothesis.

### 3.4. NETs Detection

Only a limited number of studies have addressed the direct effect of specific stimuli on the induction of NETs in vivo [38,93,107,162,204,221]. Regarding MPNs, Wolach et al. [100] found an increase in citrullinated histone H3 (H3cit) expression in the lung tissue sections of JAK2V617F mice in situ. As a matter of fact, it is rather complicated to observe the remnants of NETotic death either in vivo, in situ or ex vivo, since the NETotic death of neutrophils results in cell disintegration and the subsequent clearance of NETs remnants, in which the traps are first fragmented by DNases, followed by the removal of the trace components by phagocytic cells [222]. Therefore, they may not be observed subsequently in tissue histology [107]. However, Chrysanthopoulou et al. [168] showed the deposition of NETs components in human lung fibrotic biopsies. Although no intact neutrophils were evident in the biopsies, the remnants of NETs were observed by the extracellular localization of MPO and neutrophil elastase. The study by Berger-Achituv et al. [93] showed that tumor-associated neutrophils (neutrophils identified in the tumor bed) in the diagnostic biopsies of Ewing sarcoma, are activated to produce NETs, pointing to a possible role for NETs in cancer. We refer the readers to the article of de Buhr and von Köckritz-Blickwede [221] wherein an overview of the selected examples for in vivo and in situ detections of NETs, as well as the main NETs visualization techniques used for quantification of NETs, is given. Likewise, it is also difficult to observe the remnants of vital NET formation. Regarding vital NET formation, functional anuclear neutrophils, which retain viability and effector functions such as chemotaxis and phagocytosis, can be generated as enucleated cytoplasts [107,112]. Another option is the existence of viable neutrophils lacking mitochondria [108,223]. An anuclear neutrophil could be seen by the transmission electron microscopy of NET formation [107,159]. It is not known how these enucleated cytoplasts are removed [112]. It would be prolific to look into such structures in MPNs, either the remnants of NETs in the bone marrow biopsies or neutrophils as enucleated cytoplasts.

More accessible NET remnants are plasmatic biomarkers of NETs, such as cell-free DNA, circulating nucleosomes, MPO–DNA or NE–DNA complexes [99,101,103,113]. Although these reflect in vivo conditions, not all of them are specific for NETs, e.g., cfDNA or circulating nucleosomes. cfDNA originates from cells dying by programmed cell death, including NET formation, as well as by necrosis [101,185,222,224], because of active secretion [185,225] or as a result of tissue damage, which is a frequent phenomenon with inflammatory responses [171]. cfDNA related to nucleosomes is one of the hallmarks of apoptosis [226], but nucleosomes are also released during NET formation [113]. It has been reported that most cfDNA in the plasma of healthy individuals originates from a hematopoietic system, with minimal contributions of other tissues [227,228]. However, it is shown that the level of cfDNA and MPO–DNA complexes [101] as well as the level of circulating nucleosomes [99,103] are elevated in patients with MPNs. Tracing the cell sources of cfDNA, based on the DNA methylation patterns [228], could identify the cfDNA origin in MPNs, and consequently the neutrophil contribution to the cfDNA pool in MPNs.

## 4. Conclusions

Undeniably, it is becoming crucial to understand the molecular pathways leading to NET release given its broad implication in health and diseases. What still leaves many open questions regarding these mechanisms is the fragile and short-lived nature of neutrophils [229]. After all, this is the second decade of NET research [170], since the first detailed description of NETs happened in 2004 [38]. Likewise, this is the second decade since JAK2V617F mutation in MPN disease was detected in 2005 [5,6,7,8]. What can place NETs in the underlying pathological milieu of MPNs are MPN neutrophils and their clonality, which favors excessive inflammation and can orchestrate the evolution of a pathological microenvironment in MPNs.

## Data Availability

Not applicable.
